# Methodological quality of test accuracy studies included in systematic reviews in obstetrics and gynaecology: sources of bias

**DOI:** 10.1186/1472-6874-11-7

**Published:** 2011-03-22

**Authors:** Rachel K Morris, Tara J Selman, Javier Zamora, Khalid S Khan

**Affiliations:** 1 School of Clinical and Experimental Medicine (Reproduction, Genes and Development), University of Birmingham, Birmingham Women's Hospital, Birmingham, B15 2TG, UK; 2 Clinical Biostatistics Unit, Hospital Ramón y Cajal, Madrid, Spain

## Abstract

**Background:**

Obstetrics and gynaecology have seen rapid growth in the development of new tests with research on these tests presented as diagnostic accuracy studies. To avoid errors in judgement it is important that the methodology of these studies is such that bias is minimised. Our objective was to determine the methodological quality of test accuracy studies in obstetrics and gynaecology using the Quality Assessment of Diagnostic Accuracy Studies (QUADAS) checklist and to assess sources of bias.

**Methods:**

A prospective protocol was developed to assess the impact of QUADAS on ten systematic reviews performed over the period 2004-2007.We investigated whether there was an improvement in study quality since the introduction of QUADAS, whether a correlation existed between study sample size, country of origin of study and its quality. We also investigated whether there was a correlation between reporting and methodological quality and by the use of meta-regression analyses explored for items of quality that were associated with bias.

**Results:**

A total of 300 studies were included. The overall quality of included studies was poor (> 50% compliance with 57.1% of quality items). However, the mean compliance with QUADAS showed an improvement post-publication of QUADAS (54.9% versus 61.4% p = 0.002). There was no correlation with study sample size. Gynaecology studies published from the United States of America showed higher quality (USA versus Western Europe p = 0.002; USA versus Asia p = 0.004). Meta-regression analysis showed that no individual quality item had a significant impact on accuracy. There was an association between reporting and methodological quality (r = 0.51 p < 0.0001 for obstetrics and r = 0.56 p < 0.0001 for gynaecology).

**Conclusions:**

A combination of poor methodological quality and poor reporting affects the inferences that can be drawn from test accuracy studies. Further compliance with quality checklists is required to ensure that bias is minimised.

## Background

Obstetrics and gynaecology have seen rapid growth in the development of new tests [1-4]. For instance, tests designed to detect small for gestational age fetuses and to improve the staging of cancers have grown in recent years [5-9]. A key aspect of research on these is presented in the form of test accuracy studies [10], which generate a comparison of measurements made by an index test against those of an accepted reference standard test - the "gold standard". These comparisons enable an assessment of the accuracy of an index test, which are often expressed as sensitivity and specificity, likelihood ratios (LRs), diagnostic odds ratio (DOR), or area under a receiver-operator characteristics curve [11]. Using this information enables readers to make judgements relating to the potential suitability of new tests for clinical practice.

To avoid errors in judgement it is important that the methodology of the study is such that bias is minimised. The reporting of the study should allow for the detection of any biases by providing a complete and transparent description of the study participants, methodology and results. Guidelines for the reporting of other study types have widely been accepted e.g. CONSORT [12] for randomised control trials and QUOROM [13] and MOOSE [14] for systematic reviews. The recommended format for reporting primary accuracy evaluations of tests is called Standards for Reporting of Diagnostic Accuracy - STARD [15]. When studies of this type are incorporated in systematic reviews, assessment of their methodological quality is necessary. This allows methodological flaws, which can lead to bias, and sources of variation that might lead to heterogeneity, to be identified. An evidence based methodological quality assessment tool has been developed called Quality Assessment of Diagnostic Accuracy Studies (QUADAS) [16]. The need for quality appraisal of included studies in systematic review has been recognised for many years however, how deficiencies in study quality should be addressed in meta-analysis is not as clear [17,18].

The QUADAS initiative provides an assessment tool for the quality of test accuracy studies, as is required when using these studies in systematic reviews. It combines empirical evidence and expert opinion into a checklist of 14 quality items. As these quality items should be adhered to and then reported in a study, they are directly, and indirectly duplicated in the STARD checklist. Although gaps in reporting of quality item themselves do not necessarily mean that the methodological quality is poor, interpretation is made difficult [19]. The use of one standard checklist for assessment of study quality in all diagnostic reviews should allow clinicians to make comparable assessment of different studies. Where previous studies have attempted to assess methodological or reporting quality of test accuracy studies, a strong relationship has been found between various quality items and test accuracy results [20,21]. This study aims to assess the impact of the QUADAS initiative, on test accuracy studies, in antenatal screening and gynaecologic oncology.

## Methods

A prospective protocol was developed to assess the impact of QUADAS on ten systematic reviews performed over the period 2004-2007. These systematic reviews were selected as they were all performed by the authors, according to prospective protocols and recommended methodology, with prospective assessment of methodological quality using the QUADAS checklist thus uniform assessment could be ensured. We included reviews of minimal and non invasive tests to determine the lymph node status in gynaecological cancers [5-7] and reviews of Down's serum screening markers and uterine artery Doppler to predict small for gestational age fetuses in obstetrics [8,9]. The checklist was also tailored to take into account the nature of each review e.g. the nature of the index test (the tailored checklists are available as appendices to the published reviews). We addressed the following questions: What is the quality of studies in these fields? Is there a difference in quality between studies in Obstetrics and Gynaecology? Did the introduction of QUADAS improve quality? Does study size correlate with quality? Is there a geographical pattern to quality? Is there a relationship between compliance with STARD and QUADAS? Which quality items are associated with bias?

The QUADAS checklist was applied to each of the studies included in the reviews with the quality item being determined as either present, absent, unclear or not applicable (additional file 1). All studies were assessed in duplicate by TJS and RKM, where there was disagreement this was resolved by consensus with a third reviewer (KSK). All studies were also assessed for reporting quality using the STARD checklist. Results of individual studies were summarized in two by two tables from which the DOR was calculated as a measure of diagnostic accuracy [11]. DOR is the odds of a positive result in a diseased person relative to the odds of a positive result in a non diseased person. In the case of zero entities in the two by two tables 0.5 was added to the cells to enable calculation of DOR [22]. In the event that several tests had been applied to the same patient, the results including the largest number of patients were used in this study or where there was no difference, one index test was selected at random, this ensured patients were only included once.

The percentage compliance of studies with QUADAS items was compared between both specialties, before and after the introduction of QUADAS, using the unpaired t test to assess the effect of QUADAS on the methodological quality of studies. With the publication of QUADAS in 2003 the assumption was made that all studies published pre 2005 were published without the benefit of this directorate.

We examined the relationship between sample size and compliance with QUADAS using Spearman's rank correlation coefficient (Rho). Kruskal Wallis was used to investigate any relationship between geographical distribution and reporting quality. The country of origin of a study was determined by the country of the corresponding author. Where a significant result was found, pairways comparison was made using Conover Inman procedure. Countries were grouped depending on the number of articles published and the mean journal impact factor and adjusted for gross domestic product and population, based on previous publication [23]. Where there was a large disparity in number of studies per geographical area, some studies were re grouped to avoid large differences in group size and potentially spurious results. For obstetric reviews geographical areas were Oceania, USA, Canada, Asia, Japan, Africa, Eastern Europe and Western Europe and for gynaecology studies there were no studies from Oceania or Canada, but Latin America was added.

If the standard of reporting of a study is poor then this can potentially limit the assessment of the quality of study design. To investigate the relationship between reporting and methodological quality, the studies' compliance with STARD and QUADAS was compared using Spearman correlation coefficient. The difference in compliance with the two checklists between obstetrics and gynaecology was assessed using unpaired t test.

The final analysis performed was a meta-regression analysis to assess which quality items were associated with bias. Multiple logistic regression models were adjusted to test the effect of individual QUADAS quality items on diagnostic accuracy, measured as the diagnostic odds ratio (DOR) [24]. This methodology [25] has been used successfully in demonstrating empirically the effect of bias related to methodological flaws in clinical trials [26-28] and in diagnostic studies [29]. The dependent variable in each logistic model was a binary variable representing disease status (diseased verses non diseased) from each meta-analysis. The independent variables included a variable representing test threshold (i.e. the sum of logits of sensitivity and 1-specificity); a binary variable for test result (positive versus negative); indicator variables to control for the effect of the primary studies and the "QUADAS item (dichotomized as Yes versus all other) by test result" interaction terms to analyze its association with estimates of diagnostic accuracy. The estimated effect of a quality characteristic on average diagnostic accuracy is given by the coefficient of this latter variable whose exponentiation gives the diagnostic performance (DOR) of studies failing to satisfy the methodological criterion relative to its performance in studies with that feature. This is the Relative Diagnostic Odds Ratio (RDOR). If this ratio is greater than 1 then the accuracy of studies without that feature overestimates the diagnostic performance compared to studies with that feature. Only meta-analyses that contained studies with and without the characteristic could contribute to this estimate. We used the RDOR as the summary measure of accuracy and dependant variable in the analyses as it is useful as a single indicator of test performance.

In the initial analysis those quality items coded as unclear and not applicable were excluded. For all of the above analysis, due to the uncertainty of whether reporting items coded as unclear represented methodological failure, sensitivity analysis was performed excluding unclear as a code and adding it to the not reported group for all comparisons. Similarly sensitivity analysis was also performed to assess the effect of those items assessed as not applicable, with their initial exclusion in the analysis and then addition as if they were reported i.e. "yes" so as not to penalise studies which had a larger number of not applicable items and would therefore potentially have a seemingly lower compliance with QUADAS.

## Results

**Figure 1:**
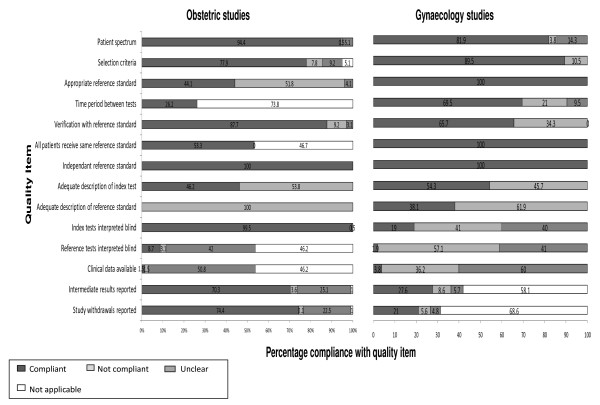
**Percentage compliance with individual QUADAS criteria for included test accuracy studies in obstetrics and gynaecology**.

A total 300 studies (195 obstetric and 105 gynaecologic studies) from ten systematic reviews were identified and included in this study. 85.6% (167/195) of the obstetric studies and 93% (98/105) of the gynaecological studies were published prior to the QUADAS initiative. The overall percentage compliance with individual quality items is shown in figure 1. The included studies for both reviews complied adequately > 50% of the time for 57.1% (8/14) of the items assessed. Items where quality was uniformly poor (both obstetrics and gynaecology < 50%) were an adequate description of the performance of the reference standard, reporting whether the reference test results were interpreted blind to the index test results and whether clinical data was available at the time of test interpretation. In addition for obstetric studies only 44.1% used an appropriate reference standard and only in 55.3% of studies did all patients receive the same reference standard. This reflects the nature of the poor quality of reference standards employed in the obstetric reviews and the lack of an accepted "gold standard" for the conditions under investigation (fetal growth restriction). In only 19% of gynaecology studies was the index test interpretation blind, reflecting the nature of the tests assessed in these reviews.

There was an improvement in the mean compliance with quality items after publication of the QUADAS checklist (54.9% versus 61.4%) which reached statistical significance (0 = 0.002); this was mainly due to an improvement in gynaecology studies (54.4% versus 70.4%) rather than obstetrics (55.5% versus 59.2%). Analysis of the correlation between sample size and QUADAS revealed no correlation for obstetrics (Rho = 0.14, p = 0.06) or gynaecology (Rho = -0.047, p = 0.64). For these analyses sensitivity analysis as described in the methods section showed no significant difference.

The mean compliance with QUADAS according to country of publication of study is shown in table 1. Investigation in to the relationship between geographical area of publication with QUADAS showed no association between compliance and area for the primary analysis in either obstetrics (p = 0.73) or gynaecology (p = 0.12). However for gynaecology, sensitivity analysis revealed a positive correlation between the compliance with QUADAS when those items considered not applicable were included with those items that had been reported (p = 0.05). Further pair-wise comparison using Conover Inman procedure showed that studies from the USA had greater compliance (USA versus Western Europe p = 0.002; USA versus Asia p = 0.004).

**Table 1:** Mean percentage compliance of studies with QUADAS according to geographical area of publication

Area of publication	Mean percentage compliance obstetrics (%) [number of studies]	Mean percentage compliance gynaecology (%) [number of studies]
Africa	50% [1]	No studies
Asia	56.3% [8]	57.7% [12]
Canada	55.1% [7]	No studies
Eastern Europe	52.7% [16]	58.6% [5]
Japan	55.1% [7]	58.9% [4]
Latin America	No studies	57.1% [2]
Oceania	61.6% [8]	35.6% [1]
United States of America	55.2% [44]	50.5% [1]
Western Europe	55.7% [104]	57.5% [52]

**Figure 2:**
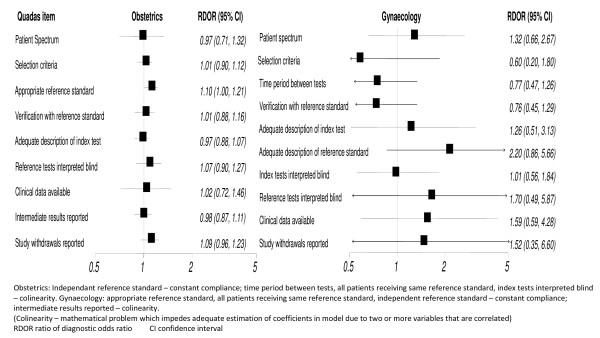
**Effect of compliance with QUADAS quality item on the ratio of the diagnostic odds ratio in studies of test accuracy in obstetrics and gynaecology**.

In the meta regression analysis for gynaecology studies initially only one of the QUADAS items had a significant impact on the diagnostic accuracy of the studies and that was whether a manuscript explained the withdrawals from a study. In those studies where withdrawals were not explained there was an overestimation in the accuracy of the test (p = 0.005). However, in the majority of studies this quality item was coded as 'not applicable', thus when the analysis was repeated with these studies removed, adherence to this quality item also failed to have an impact on test accuracy. In the meta-regression for obstetrics, only QUADAS item 3 (appropriate reference standard) had a marginal impact on diagnostic accuracy (p = 0.05), so that studies in which an inappropriate reference standard was used overestimated the diagnostic accuracy by 10%. The results are illustrated in figure 2.

**Figure 3:**
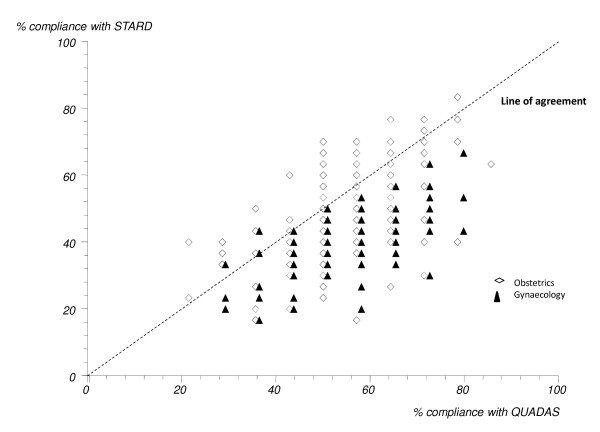
**Scatter plot showing the level of agreement between the percentage compliance of test accuracy studies in obstetrics and gynaecology with the STARD and QUADAS check lists**.

All included papers were assessed for reporting standard and overall this was poor. The included obstetric studies reported adequately > 50% of the time for 62.1% (18/29) of the items as assessed in this review and for gynaecology 51.7% (15/29). Only 2 obstetric papers (no gynaecology papers) used a STARD flow diagram and these were published after the publication of the STARD statement. There was significant correlation between the percentage compliance of studies with STARD and QUADAS checklists for obstetrics (Rho = 0.51, p = < 0.0001) and gynaecology (Rho 0.56, p = < 0.0001) which is illustrated in figure 3. This figure shows that when studies had a higher standard of reporting they also had a higher standard of methodology.

## Discussion

This study showed that there was an improvement in the methodological quality of test accuracy studies in gynaecology cancer since the introduction of the QUADAS initiative but not for obstetrics. Unsurprisingly, due to the overlap in quality items between the two checklists there was a positive correlation between compliance with STARD and QUADAS. Sample size showed no correlation with compliance. Studies from the USA had greater compliance with QUADAS for gynaecology studies. No correlation with geographical area was seen for obstetrics. Meta regression did not show any significant correlation between compliance with QUADAS item and test accuracy.

The strengths of our study lie in the large number of included studies and meta-analyses, the continuity in assessment using the same two reviewers throughout and the use of tailored checklists to take into account the differences in studies in gynaecological oncology and obstetrics (e.g. the utilisation of the not applicable category). Limitations to our study include the small proportion of included studies that were reported after publication of the QUADAS tool and the overall poor reporting standard of the included papers. As a true assessment of a study's methodological quality relies on good reporting, we have to conclude that the poor methodological quality of the papers in this review may actually reflect a combination of poor study design as well as poor reporting. Our investigation into the effect of individual items of study quality on diagnostic accuracy could find no significant relationship between any individual quality item and accuracy. Although we could demonstrate an improvement in methodological quality since the introduction of QUADAS we cannot conclude that this improvement is due to the QUADAS initiative or due to other factors such as a historical progression in improved methodological techniques.

## Conclusion

We would recommend that all future test accuracy studies adhere to the QUADAS guidelines and that when studies are being included in systematic reviews, reviewers must assess for reporting and methodological quality using the QUADAS items that are relevant to their study area and consider additional items where necessary. As adherence to QUADAS becomes more widespread, the effect of items of methodological quality on diagnostic accuracy should be reassessed to enable clinicians to interpret the validity and generalisability of results. This type of research will also help to improve test accuracy study design.

## Competing interests

The authors declare that they have no competing interests.

## Authors' contributions

The following authors were responsible for study concept and design: TJS, RKM, JZ, KSK. TJS and RKM take responsibility for acquisition of data. All authors were responsible for analysis, interpretation of data, drafting of the manuscript, critical revision of the manuscript and statistical analysis. All authors confirm that they have read and approved the final manuscript.

## Pre-publication history

The pre-publication history for this paper can be accessed here:

http://www.biomedcentral.com/1472-6874/11/7/prepub

## Supplementary Material

Additional file 1 **Supplemental file 1 - QUADAS checklist**. The quality assessment of studies of diagnostic accuracy checklist with description of checklist items. Click here for file
