# Supplementary material for: Methodological quality of test accuracy studies included in systematic reviews in obstetrics and gynaecology: sources of bias
Source: BMC Womens Health. 2011 Mar 22;11:7. doi: 10.1186/1472-6874-11-7 (PMC3072918; doi:10.1186/1472-6874-11-7)
Supplement: Additional file 1 — Supplemental file 1 - QUADAS checklist. The quality assessment of studies of diagnostic accuracy checklist with description of checklist items. [file 1472-6874-11-7-S1.DOC]

Supplemental file S1: Quadas checklist

|  | **QUADAS Item** | **Code 1 2 3 4** |
| --- | --- | --- |
| Was the spectrum of patients representative of the patients who will receive the test in practice? | **1** | Yes □ No □ Unclear □ NA □ |
| Were selection criteria clearly described? | **2** | Yes □ No □ Unclear □ NA □ |
| Is the reference standard likely to correctly classify the target condition? | **3** | Yes □ No □ Unclear □ NA □ |
| Is the time period between reference standard and index test short enough to be reasonably sure that the target condition did not change between the two tests? | **4** | Yes □ No □ Unclear □ NA □ |
| Did the whole study population or a random selection of the sample, receive verification using a reference standard for diagnosis? | **5** | Yes □ No □ Unclear □ NA □ |
| Did patients receive the same reference standard regardless of the index test result? | **6** | Yes □ No □ Unclear □ NA □ |
| Was the reference standard independent of the index test? | **7** | Yes □ No □ Unclear □ NA □ |
| Was the execution of the index test described in sufficient detail to permit replication of the test? | **8** | Yes □ No □ Unclear □ NA □ |
| Was the execution of the reference standard described in sufficient detail to permit its replication? | **9** | Yes □ No □ Unclear □ NA □ |
| Were the index test results interpreted without the knowledge of the results of the reference standard? | **10** | Yes □ No □ Unclear □ NA □ |
| Were the reference standard results interpreted without knowledge of the index test results? | **11** | Yes □ No □ Unclear □ NA □ |
| Were the same clinical data available when test results were interpreted as would be available when the test is used in practice? | **12** | Yes □ No □ Unclear □ NA □ |
| Were uninterpretable / intermediate test results reported? | **13** | Yes □ No □ Unclear □ NA □ |
| Were withdrawals from the study explained? | **14** | Yes □ No □ Unclear □ NA □ |
